# Prognostic value of tumor infiltrating NK cells and macrophages in stage II+III esophageal cancer patients

**DOI:** 10.18632/oncotarget.12484

**Published:** 2016-10-05

**Authors:** Bin Xu, Lujun Chen, Jing Li, Xiao Zheng, Liangrong Shi, Changping Wu, Jingting Jiang

**Affiliations:** ^1^ Department of Tumor Biological Treatment, The Third Affiliated Hospital of Soochow University, Jiangsu Changzhou 213003, China; ^2^ Research Center for Cancer Immunotherapy Technology of Jiangsu Province, The Third Affiliated Hospital of Soochow University, Jiangsu Changzhou 213003, China; ^3^ Department of Oncology, The Third Affiliated Hospital of Soochow University, Jiangsu Changzhou 213003, China

**Keywords:** tumor microenvironment, tumor infiltrating immune cells, NK cells, macrophages, esophageal cancer

## Abstract

The detailed understanding of the immunobiology of tumor microenvironment has recently translated into new therapeutic approach against human cancers. Besides the role of immune cells mediating adaptive immune responses, the tumor infiltrating components of the innate immune system including, neutrophils, mast cells, NK cells, and macrophages, also role importantly in anti-tumor immunity. In our present study, we retrospectively analyzed the prognostic value of the densities of tumor infiltrating NK cells and macrophages in esophageal cancer tissues derived from stage II+III patients. Our results showed that the density of the infiltrating NK cells in tumor stroma was significantly associated with nodal status. In addition, the densities of the infiltrating NK cells in tumor nest, and the infiltrating macrophages in tumor nest as well as in tumor stroma, were significantly associated with patients' postoperative prognoses. Furthermore, the combination of infiltrating NK cells in tumor nest and stroma, or the combination of infiltrating macrophages in tumor nest and stroma, could also be used as important prognostic tool in predicting the survival of the stage II+III esophageal cancer patients.

## INTRODUCTION

Esophageal cancer is one of the most important cancer types of human digestive tract, and still has a high rate of incidence and mortality in China [1]. Many factors have been shown to be associated with its progression including local inflammation and esophagitis, in addition to some other habits such as intake of alcohol, tobacco, very hot drinks, and poor diet [2]. It is categorized mainly into two sub-types, a) esophageal squamous-cell carcinoma (ESCC) and, b) esophageal adenocarcinoma (EAC) [3]. The ESCC seems to be more common in the developing world, while the EAC is more prevalent in the developed world [4]. Despite the use of numerous therapeutic strategies such surgery, chemotherapy, radiotherapy, immunotherapy and other combined therapies for its clinical treatment, the 5-year survival still remains poor [5]. Thus, it reinforces the need to identify and establish, additional novel prognostic tools for the clinical evaluation of patients suffering from esophageal cancer.

The immune suppression of the tumors by immune cells and molecules of the tumor microenvironment has drawn much attention and recently shown promising immunotherapeutic results in various cancers [6]. We have also previously reported about the anti-tumor immunity role of adaptive T-cells, importantly, in the progression and prognostic prediction of human esophageal cancer [7, 8]. We showed that increased infiltrating densities of T-bet^+^ lymphocytes and CD8^+^ T cells, positively associated, while increased infiltrating density of Foxp3^+^ Tregs, negatively associated with better postoperative prognosis of esophageal cancer patients, [8]. Moreover, beside the immune cells mediating adaptive immune response, the tumor infiltrating innate immune cells like neutrophils, mast cells, NK cells, macrophages also contributed towards an anti-tumor immunity function [9–12]. In this regard, Chaput *et al.* [11] have reported that the infiltrating density of CD57^+^ nature killer (NK) cells and CD68^+^ macrophages, could be used as an independent prognostic predictor of stage II and III, human colorectal cancer, respectively. This suggested that these molecules can be important prognostic predictors and could be routinely used in patients suffering from this malignancy.

In the present study, we retrospectively analyzed the prognostic value of the varying densities of tumor infiltrating NK cells or macrophages in human esophageal cancer at stage II+III. In addition we also tried to understand the link between the combined infiltration of NK cells and macrophages and death risk of esophageal cancer patients.

## RESULTS

### Correlation of CD57^+^ NK cells and CD68^+^ macrophages infiltration in the tumor nest and stroma, with patient's characteristics

**Figure 1:**
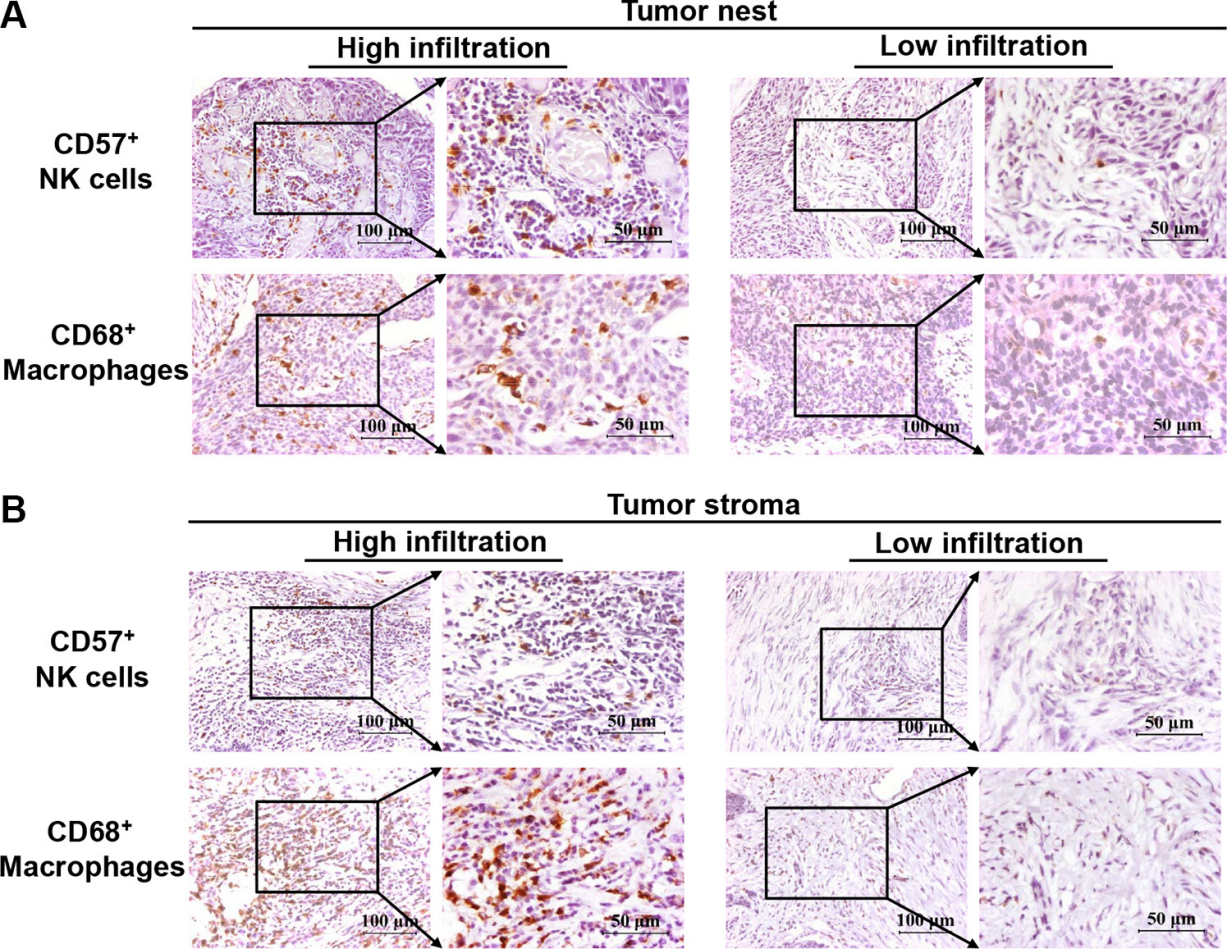
Immunohistochemical analysis of CD57^+^ NK cells and CD68^+^ macrophages in esophageal cancer tissues from stage II+III patients Panel (**A**) depicts the high and low infiltration of CD57^+^ NK cells and CD68^+^ macrophages in tumor nests, while panel (**B**) depicts the high and low infiltration of CD57^+^ NK cells and CD68^+^macrophages in tumor stroma.

The characteristics of the 138 eligible patients are shown in Tables 1 and 2. The data showed that 90 patients had high infiltrating NK cells, while 70 patients had high infiltrating macrophages in the tumor nest. In contrast, 48 patients had low infiltrating NK cells and 68 patients had low infiltrating macrophages in tumor stroma. Except for the correlation between nodal status and infiltrating density of CD57^+^ NK cells in the tumor stroma, no other significant correlation was observed between infiltrating density of CD57^+^ NK cells, CD68^+^ macrophages and clinical parameters, such as gender, age, tumor size and TNM stage in tumor nest and stroma. High and low infiltrating densities of CD57^+^ NK cells and CD68^+^ macrophages were shown in Figure 1.

**Table 1:** Correlation between clinical parameters and the infiltrating density of CD57^+^ NK cells in esophageal cancer tissues

Clinical parameters	Cases	CD57^+^ NK cells in Tumor Nest					CD57^+^ NK cells in Tumor Stroma				
		Low		High		*P*-value	Low		High		*P*-value
		*n*	%	*n*	%		*n*	%	*n*	%	
Gender											
Male	102	33	32.35	69	67.65	0.313	63	61.76	39	38.24	0.717
Female	36	15	41.67	21	58.33		21	58.33	15	41.67	
Age (years)											
< 60	73	23	31.51	50	68.49	0.392	42	57.53	31	42.47	0.395
≥ 60	65	25	38.46	40	61.54		42	64.62	23	35.38	
Tumor size (cm)											
≤ 3.5	56	17	30.36	39	69.64	0.367	35	62.50	21	37.50	0.746
> 3.5	82	31	37.80	51	62.20		49	59.76	33	40.24	
Tumor (T) status^a^											
pT_1_	6	4	66.67	2	33.33	0.384	4	66.67	2	33.33	0.804
pT_2_	65	22	33.85	43	66.15		37	56.92	28	43.08	
pT_3_	54	17	31.48	37	68.52		34	62.96	20	37.04	
pT_4_	13	5	38.46	8	61.54		9	69.23	4	30.77	
Nodal (N) status^b^											
N_0_	84	30	35.71	54	64.29	0.774	58	69.05	26	30.95	**0.014**
N_1_	54	18	33.33	36	66.67		26	48.15	28	51.85	
TNM stage											
II	101	35	34.65	66	65.35	0.958	62	61.39	39	38.61	0.837
III	37	13	35.14	24	64.86		22	59.46	15	40.54	
Total	138										

Values in bold signify *P* < 0.05.

a Tumor status is classified as follows: pT_1_, invasion of lamina propria or submucosa; pT_2_, invasion of muscularis propria; pT_3_, invasion of adventitia; and pT_4_, invasion of adjacent structures.

b Nodal status is classified as follows: N_0_, no regional lymph-node metastasis; N_1_, regional lymph-node metastasis.

**Table 2:** Correlation between clinical parameters and the infiltrating density of CD68^+^ macrophages in esophageal cancer tissues

Clinical parameters	Cases	CD68^+^ macrophages in Tumor Nest					CD68^+^ macrophages in Tumor Stroma				
		Low		High		*P*-value	Low		High		*P*-value
		*n*	%	*n*	%		*n*	%	*n*	%	
Gender											
Male	102	50	49.02	52	50.98	0.919	71	69.61	31	30.39	0.540
Female	36	18	50.00	18	50.00		27	75	9	25.00	
Age (years)											
< 60	73	37	50.68	36	49.32	0.726	22	30.14	51	69.86	0.752
≥ 60	65	31	47.69	34	52.31		18	27.69	47	72.31	
Tumor size (cm)											
≤ 3.5	56	30	53.57	26	46.43	0.404	17	41.46	39	95.12	0.769
>3.5	82	38	46.34	44	53.66		23	23.71	59	60.82	
Tumor (T) status^a^											
pT_1_	6	1	16.67	5	83.33	0.900	1	16.67	5	83.33	0.509
pT_2_	65	34	52.31	31	47.69		19	29.23	46	70.77	
pT_3_	54	28	51.85	26	48.15		15	27.78	39	72.22	
pT_4_	13	5	38.46	8	61.54		5	38.46	8	61.54	
Nodal (N) status^b^											
N_0_	84	46	54.76	38	45.24	0.108	27	32.14	57	67.86	0.308
N_1_	54	22	40.74	32	59.26		13	24.07	41	75.93	
TNM stage											
II	101	50	49.50	51	50.50	0.929	29	28.71	72	71.29	0.907
III	37	18	48.65	19	51.35		11	29.73	26	70.27	
Total	138										

a,b The same description as in Table 1.

### Univariate survival analysis based on the different combinations of CD57^+^ NK cells and CD68^+^ macrophages in tumor nest and stroma

**Figure 2:**
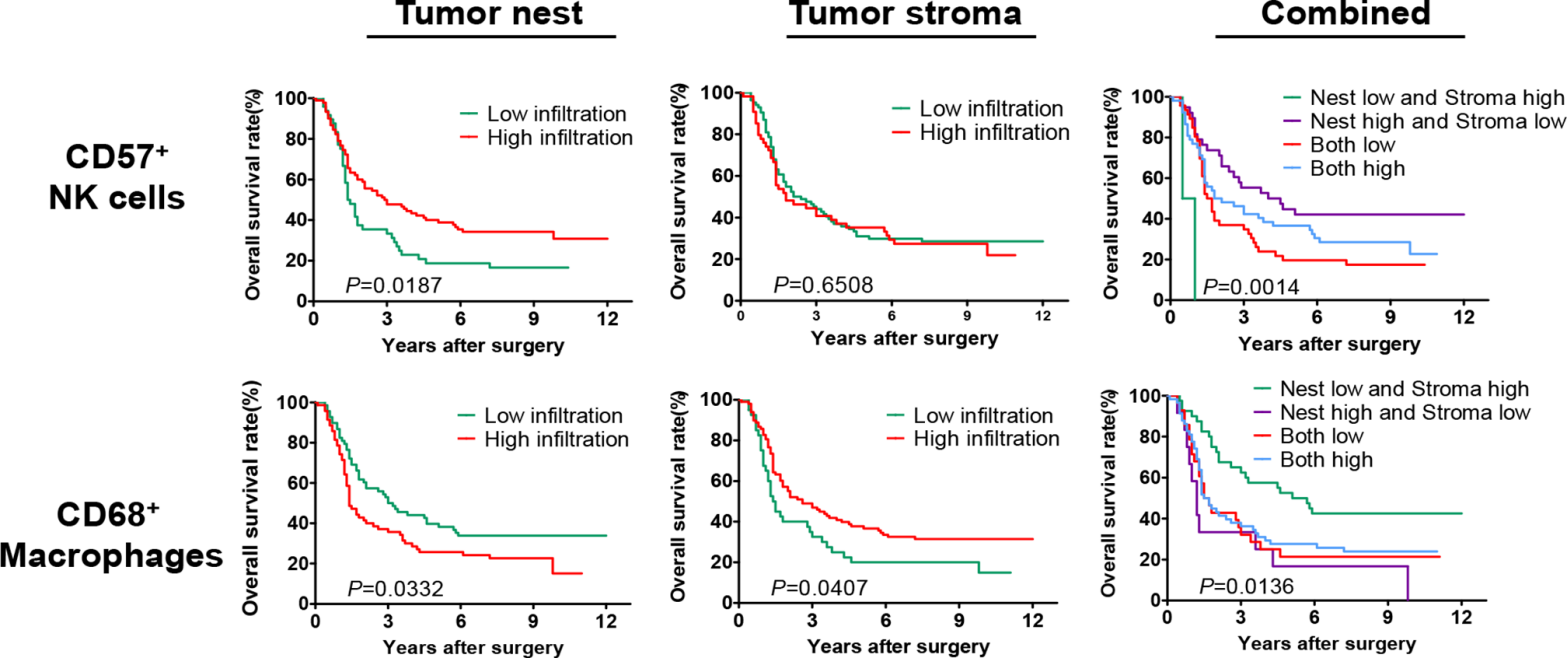
Survival analysis of the infiltrating densities of CD57^+^ NK cells and CD68^+^ macrophages in esophageal cancer tissues from stage II+III patients Patients with high infiltration density of CD57^+^ NK cells in tumor nest had a significantly better OS than those with low infiltrating (*P* = 0.0187), but there was no significant difference between high and low CD57^+^ NK cells infiltrating in tumor stroma (*P* = 0.6508). Patients with high infiltration density of CD68^+^ macrophages in tumor nest had a significantly worse OS than those with low infiltrating (*P* = 0.0332), but a higher infiltrating density of CD68^+^ macrophages in tumor stroma significantly correlated with a better OS (*P* = 0.0407). Patients with high infiltration density of CD57^+^ NK cells in tumor nest and low infiltration density in tumor stroma had a best OS, both high or both low infiltrating in tumor nest and stroma had a medium OS and the other patients with low infiltration density of CD57^+^ NK cells in tumor nest and high infiltrating density in tumor stroma had a worst OS (*P* = 0.0014). Patients with low infiltrating density of CD68^+^macrophages in tumor nest and high infiltrating density in tumor stroma had a best OS, both high infiltration in tumor nest and stroma and both low infiltration in tumor nest and stroma had the medium OS and the patients with high infiltrating density of CD68^+^ macrophages in tumor nest and low infiltrating density in tumor stroma had a worst OS (*P* = 0.0136).

As shown in Figure 2, patients with high infiltrating density of CD57^+^ NK cells in the tumor nest had significantly better overall survival (OS) than those with low infiltration (median OS: 2.95 *vs.* 1.45 years, *P* = 0.0187). However, no such significant difference was observed for OS between high and low CD57^+^ NK cells infiltration in the tumor stroma (median OS: 1.80 *vs*. 2.25 years, *P* = 0.6508). Moreover, the patients with high infiltrating density of CD68^+^ macrophages in the tumor nest had a significantly worse OS than those with low infiltration (median OS: 1.40 *vs*. 3.10 years, *P* = 0.0332). Instead, a reverse result was observed in tumor stroma, a higher infiltrating density of CD68^+^ macrophages correlated with a better OS (median OS: 2.60 *vs*. 1.45 years, *P* = 0.0407).

Furthermore, the comparison of different combinations of CD57^+^ NK cells infiltration between tumor nest and stroma showed that patients with high infiltrating density of CD57^+^ NK cells in tumor nest and low infiltrating density in tumor stroma had a best OS, while high or low infiltration in both the tumor nest and stroma displayed medium OS respectively, and the patients with low infiltrating density of CD57^+^ NK cells in tumor nest and high infiltrating density in tumor stroma had a worst OS (median OS: 4.25, 1.95, 1.60 and 0.75 years, *P* = 0.0014). Similarly, the correlation of different combinations of CD68^+^ macrophages infiltrating between tumor nest and stroma with OS suggested that, patients with low infiltrating density of CD68^+^ macrophages in tumor nest and high infiltration density in tumor stroma had a best OS, while high or low infiltration in the both tumor nest and stroma displayed medium OS, and the patients with high infiltrating density of CD68^+^ macrophages in tumor nest and low infiltrating density in tumor stroma had a worst OS (median OS: 5.40, 1.60, 1.60 and 1.20 years, *P* = 0.0136).

**Figure 3:**
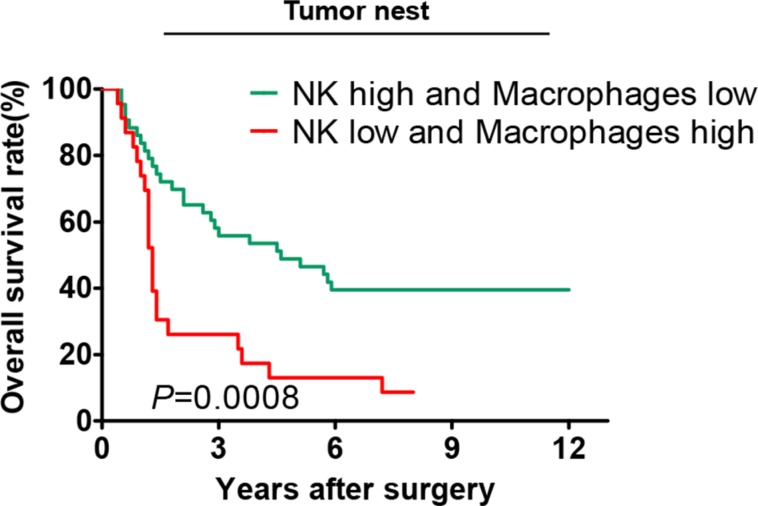
Correlation of the overall survival rate with different combinations of CD57^+^ NK cells and CD68^+^ macrophages in the tumor nests Only patients with high infiltrating density of CD57^+^ NKcells and low infiltrating density of CD68^+^macrophages in tumor nest and the patients with low infiltrating density of CD57^+^ NKcells and high infiltrating density of CD68^+^macrophages in tumor nest were included for this survival analysis.

As shown in Figure 3, patients with high infiltrating density of CD57^+^ NK cells and low infiltrating density of CD68^+^ macrophages in tumor nest had a significantly better OS compared with the patients with low infiltrating density of CD57^+^ NK cells and high infiltrating density of CD68^+^ macrophages (median OS: 4.60 *vs*. 1.30 years, *P* = 0.0008).

### Multivariate survival analysis based on the different combinations of CD57^+^ NK cells and CD68^+^ macrophages in tumor nest and stroma

As shown in Table 3, patients with high infiltrating density of CD57^+^ NK cells in tumor nest had significant reduced death risk (HR = 0.597, 95% CI: 0.392–0.908, *P* = 0.016) compared with the reference group (patients with low infiltrating density of CD57^+^ NK cells) after adjusting for gender, age, tumor size, TNM stage and treatment. however, high infiltrating density of CD57^+^ NK cells in tumor stroma, did not show any significant change in the death risk (HR = 1.015, 95% CI: 0.670–1.536, *P* = 0.945) compare with the patients with low infiltration, after adjusting for gender, age, tumor size, TNM stage and treatment, as shown in Table 4.

**Table 3:** Cox model analysis for the correlation between the infiltrating density of CD57^+^ NK cells in tumor nest and hazard ratio (*n* = 138)

Clinical parameters	Univariate			Multivariate		
	HR	95% CI	*P*-value	HR	95% CI	*P*-value
Gender						
Male/Female	0.982	0.682−1.536	0.937	0.986	0.615−1.579	0.952
Age (years)						
≥ 60/< 60	0.930	0.627−1.378	0.717	0.756	0.497−1.152	0.193
Tumor size (cm)						
≥ 3.5/< 3.5	1.417	0.941−2.132	0.095	1.431	0.920−2.227	0.112
TNM Stage						
S_III_/S_II_	1.310	0.850−2.019	0.220	1.211	0.768−1.911	0.410
Treatment						
Chemotherapy/None	1.132	0.610−2.098	0.695	0.893	0.464−1.718	0.735
Radiotherapy/ None	0.651	0.398−1.064	0.087	0.569	0.342−0.946	**0.030**
Both/None	0.658	0.091−4.743	0.678	0.681	0.089−5.201	0.681
NK cells in tumor nest						
High/Low	0.614	0.410−0.920	**0.018**	0.597	0.392−0.908	**0.016**

Values in bold signify *P* < 0.05.

**Table 4:** Cox model analysis for the correlation between the infiltrating density of CD57^+^ NK cells in tumor stroma and hazard ratio (*n* = 138)

Clinical parameters	Univariate			Multivariate		
	HR	95% CI	*P*-value	HR	95% CI	*P*-value
Gender						
Male/Female	0.982	0.682−1.536	0.937	0.926	0.580−1.479	0.749
Age (years)						
≥ 60/< 60	0.930	0.627−1.378	0.717	0.825	0.546−1.248	0.362
Tumor size (cm)						
≥ 3.5/< 3.5	1.417	0.941−2.132	0.095	1.466	0.943−2.277	0.089
TNM Stage						
S_III_/S_II_	1.310	0.850−2.019	0.220	1.220	0.777−1.915	0.388
Treatment						
Chemotherapy/None	1.132	0.610−2.098	0.695	0.968	0.506−1.851	0.922
Radiotherapy/ None	0.651	0.398−1.064	0.087	0.596	0.360−0.988	**0.045**
Both/None	0.658	0.091−4.743	0.678	0.651	0.085−4.955	0.680
NK cells in tumor stroma						
High/Low	1.096	0.735−1.636	0.653	1.015	0.670−1.536	0.945

Values in bold signify *P* < 0.05.

Further analysis of infiltrating densities of CD57^+^ NK cells in tumor nest and stroma based on different subgroups: “nest high and stroma low”, “nest low and stroma high”, “both low in tumor nest and stroma”, and “both high in tumor nest and stroma”. Cox model analysis for these four subgroups revealed that: patients in subgroup “nest high and stroma low” had a lowest death risk (HR = 0.105, 95% CI: 0.023–0.485, *P* = 0.004), and the death risks of patients in “both low” and “both high” subgroups were moderate(HR = 0.211, 95% CI: 0.047–0.954, *P* = 0.043 and HR = 0.152, 95% CI: 0.034–0.681, *P* = 0.014), these three subgroups all compared with the patients in subgroup “nest low and stroma high”(Table 5).

**Table 5:** Prognostic value of the combination based on infiltrating density of CD57^+^ NK cells in tumor nest and in tumor stroma in Cox proportional hazards mode analysis (*n* = 138)

Clinical parameters	Univariate			Multivariate		
	HR	95% CI	*P*-value	HR	95% CI	*P*-value
Gender						
Male/Female	0.982	0.682−1.536	0.937	1.046	0.645−1.698	0.854
Age (years)						
≥ 60/< 60	0.930	0.627−1.378	0.717	0.795	0.522−1.213	0.288
Tumor size (cm)						
≥ 3.5/< 3.5	1.417	0.941−2.132	0.095	1.332	0.852−2.082	0.209
TNM Stage						
S_III_/S_II_	1.310	0.850−2.019	0.220	1.247	0.788−1.973	0.346
Treatment						
Chemotherapy/None	1.132	0.610−2.098	0.695	0.939	0.486−1.812	0.851
Radiotherapy/ None	0.651	0.398−1.064	0.087	0.609	0.366−1.014	0.056
Both/None	0.658	0.091−4.743	0.678	0.800	0.103−6.227	0.831
NK cells in tumor nest and stroma						
Nest high and Stroma low /Nest low and Stroma high	0.079	0.018−0.353	**0.001**	0.105	0.023−0.485	**0.004**
Both low /Nest low and Stroma high	0.160	0.037−0.687	**0.014**	0.211	0.047−0.954	**0.043**
Both high /Nest low and Stroma high	0.122	0.028−0.527	**0.005**	0.152	0.034−0.681	**0.014**

Values in bold signify *P* < 0.05.

In addition, after adjusting for gender, age, tumor size, TNM stage and treatment, the death risk was increased by 48.8% (HR = 1.488, 95% CI: 0.996–2.221, *P* = 0.052) in patients with high infiltrating density of CD68^+^ macrophages in the tumor nest compared with patients with low infiltrating density of these macrophages, as shown in Table 6. Conversely, high infiltrating density of CD68^+^ macrophages in tumor stroma indicated a decreased death risk (HR = 0.586, 95% CI: 0.378–0.908, *P* = 0.017) compared with patients with low infiltrating density (Table 7).

**Table 6:** Cox model analysis for the correlation between the infiltrating density of CD68^+^ macrophages in tumor nest and hazard ratio (*n* = 138)

Clinical parameters	Univariate			Multivariate		
	HR	95% CI	*P*-value	HR	95% CI	*P*-value
Gender						
Male/Female	0.982	0.682−1.536	0.937	0.912	0.574−1.449	0.697
Age (years)						
≥ 60/< 60	0.930	0.627−1.378	0.717	0.842	0.559−1.269	0.412
Tumor size (cm)						
≥ 3.5/< 3.5	1.417	0.941−2.132	0.095	1.466	0.944−2.277	0.089
TNM Stage						
S_III_/S_II_	1.310	0.850−2.019	0.220	1.170	0.744−1.839	0.497
Treatment						
Chemotherapy/None	1.132	0.610−2.098	0.695	0.935	0.491−1.780	0.837
Radiotherapy/ None	0.651	0.398−1.064	0.087	0.599	0.363−0.987	**0.044**
Both/None	0.658	0.091−4.743	0.678	0.823	0.106−6.380	0.852
CD68^+^ macrophages in tumor nest						
High/Low	1.541	1.038−2.289	**0.032**	1.488	0.996−2.221	0.052

Values in bold signify *P* < 0.05.

**Table 7:** Cox model analysis for the correlation between the infiltrating density of CD68^+^ macropahges in tumor stoma and hazard ratio (*n* = 138)

Clinical parameters	Univariate			Multivariate		
	HR	95% CI	*P*-value	HR	95% CI	*P*-value
Gender						
Male/Female	0.982	0.682−1.536	0.937	0.894	0.563−1.420	0.635
Age (years)						
≥ 60/< 60	0.930	0.627−1.378	0.717	0.802	0.534−1.206	0.289
Tumor size (cm)						
≥ 3.5/< 3.5	1.417	0.941−2.132	0.095	1.410	0.908−2.192	0.126
TNM Stage						
S_III_/S_II_	1.310	0.850−2.019	0.220	1.222	0.778−1.920	0.384
Treatment						
Chemotherapy/None	1.132	0.610−2.098	0.695	1.096	0.568−2.115	0.784
Radiotherapy/ None	0.651	0.398−1.064	0.087	0.544	0.327−0.904	**0.019**
Both/None	0.658	0.091−4.743	0.678	0.692	0.090−5.290	0.722
CD68^+^ macrophages in tumor stroma						
High/Low	0.651	0.429−0.990	**0.045**	0.586	0.378–0.908	**0.017**

Values in bold signify *P* < 0.05.

As same as in the analysis of infiltrating densities of CD57^+^ NK cells in tumor nest and stroma, four subgroups of CD68^+^ macrophages in tumor nest and stroma were also defined as “nest high and stroma low”, “nest low and stroma high”, “both low in tumor nest and stroma”, and “both high in tumor nest and stroma”. Cox model analysis for these four subgroups revealed that: patients in subgroup “nest high and stroma low” had a highest death risk (HR = 3.236, 95% CI: 1.541–6.794, *P* = 0.002), and the death risks of patients in “both low” and “both high” subgroups were moderate(HR = 2.211, 95% CI: 1.210–4.075, *P* = 0.010 and HR = 1.872, 95% CI: 1.118–3.133, *P* = 0.017), and these three subgroups all compared with the patients in subgroup “nest low and stroma high”(Table 8).

**Table 8:** Prognostic value of the combination base on infiltrating density of CD68^+^ macrophages in tumor nest and in tumor stroma in COX proportional hazards mode analysis (*n* = 138)

Clinical parameters	Univariate			Multivariate		
	HR	95% CI	*P*-value	HR	95% CI	*P*-value
Gender						
Male/Female	0.982	0.682−1.536	0.937	0.869	0.541−1.394	0.560
Age (years)						
≥ 60/< 60	0.930	0.627−1.378	0.717	0.820	0.545−1.232	0.339
Tumor size (cm)						
≥ 3.5/< 3.5	1.417	0.941−2.132	0.095	1.405	0.901−2.192	0.134
TNM Stage						
S_III_/S_II_	1.310	0.850−2.019	0.220	1.156	0.733−1.822	0.533
Treatment						
Chemotherapy/None	1.132	0.610−2.098	0.695	1.073	0.556−2.071	0.835
Radiotherapy/ None	0.651	0.398−1.064	0.087	0.539	0.325−0.893	**0.017**
Both/None	0.658	0.091−4.743	0.678	1.021	0.130−8.033	0.984
CD68^+^ macrophages in nest and stroma						
Nest high and Stroma low /Nest low and Stroma high	2.913	1.416−5.995	**0.004**	3.236	1.541−6.794	**0.002**
Both low /Nest low and Stroma high	2.006	1.116−3.607	**0.020**	2.221	1.210−4.075	**0.010**
Both high /Nest low and Stroma high	1.906	1.149−3.161	**0.013**	1.872	1.118−3.133	**0.017**

Values in bold signify *P* < 0.05.

Based on different infiltrating densities of CD57^+^ NK cells and CD68^+^macrophages in tumor nest, patients were divided into four subgroups and analyzed by a Cox model. Results showed that patients with low infiltrating density of CD57^+^ NK cells and high infiltrating density of CD68^+^ macrophages correlated with a higher death risk (HR = 2.712, 95% CI: 1.492–4.930, *P* = 0.001) compared with patients with high infiltrating density of CD57^+^ NK cells and low infiltrating density of CD68^+^ macrophages (Table 9).

**Table 9:** Prognostic value of the combination based on infiltrating densities of CD57^+^ NK cells and CD68^+^ macrophages in cancer tissues in COX proportional hazards mode analysis (*n* = 138)

Clinical parameters	Univariate			Multivariate		
	HR	95% CI	*P*-value	HR	95% CI	*P*-value
Gender						
Male/Female	0.982	0.682−1.536	0.937	0.973	0.604−1.567	0.910
Age (years)						
≥ 60/< 60	0.930	0.627−1.378	0.717	0.790	0.517−1.205	0.274
Tumor size (cm)						
≥ 3.5/< 3.5	1.417	0.941−2.132	0.095	1.404	0.899−2.194	0.136
TNM Stage						
S_III_/S_II_	1.310	0.850−2.019	0.220	1.215	0.758−1.947	0.418
Treatment						
Chemotherapy/None	1.132	0.610−2.098	0.695	0.849	0.440−1.638	0.625
Radiotherapy/ None	0.651	0.398−1.064	0.087	0.581	0.350−0.963	**0.035**
Both/None	0.658	0.091−4.743	0.678	0.840	0.107−6.592	0.868
Combined with NK cells and macrophages						
CD57 low and CD68 high /CD57 high and CD68 low	2.761	1.541−4.946	**0.001**	2.712	1.492−4.930	**0.001**
Both low /CD57 high and CD68 low	1.567	0.865−2.838	0.139	1.617	0.870−3.007	0.129
Both high /CD57 high and CD68 low	1.514	0.907−2.525	0.112	1.467	0.867−2.484	0.153

Values in bold signify *P* < 0.05.

## DISCUSSION

The chronic inflammation in the tumor microenvironment is an essential aspect of cancer initiation and progression, and therefore regarded as a hallmark of cancer [13]. In the physio-pathological process of inflammation during cancer progression, many immune cells, especially the innate immune cells, including NK cells, macrophages, dendritic cells, and neutrophils has been shown to be involved not only in the prevention of tumor initiation or progression, but also in the promotion of malignant transformation and metastasis [14]. Thus, it's of great importance to better understand the role of innate immune system in anti-tumor immunity, to establish the predicting model of cancer progression, its prognosis, and develop novel therapeutic strategy based on immune intervention [15, 16]. In the present study, we focused on the clinical significance and prognostic value of tumor infiltrating NK cells and macrophages in the tumor tissues from patients suffering from stage II+III esophageal cancer.

The NK cells are a cohort of lymphoid cells which are presumed to be major innate effector cells, and role importantly in the control of cancer initiation and progression due to their lytic function [17, 18]. The activated NK cells eliminate the tumor cells by releasing perforin and granzymes, expressing FasL and TRAIL, secreting IFN-γ, and antibody-dependent cellular cytotoxicity [18]. Thus, the sufficient presence of NK cells in the tumor microenvironment and their sustained effector function, contribute essentially to the antitumor immune response and finally leads to the prevention of cancer progression and metastasis [19, 20]. Ishigami *et al.* [21] reported that the gastric cancer patients with a high density of NK cells infiltration had better prognosis than those with a low level of NK cells infiltration. Similarly, Villegas *et al.* [22] also reported that, the NK cells infiltrating density in squamous cell lung carcinoma tissues was significantly associated with patient's age and tumor stage, and could be a useful prognostic factor in this malignancy. In addition, it has also been suggested that in patients suffering from stage II+III colorectal cancer, the infiltrating densities of CD57^+^ NK cells and CD68^+^ macrophages in cancer tissues, could be used as a quick, inexpensive, and well-established method in predicting survival of those cancer patients [11].

Consistent with these observations, our data also showed that the density of the infiltrating CD57^+^ NK cells in the tumor stroma was positively and significantly associated with nodal status. The survival analysis suggested that the prognosis of patients with low infiltrating density of NK cells in tumor nest was significantly poor than of patients with high infiltration. The Cox model analysis based on the selection of gender, age, tumor size, TNM stage, treatment and NK cells infiltration specifically in tumor nest showed that the density of infiltrating NK cells could be used as an independent prognostic risk factor in predicting stage II+III esophageal cancer patients (Table 3), but this was not the case in the tumor stroma (Table 4). The combining of the densities of infiltrating NK cells in the tumor nest and stroma suggested that this parameter is again significantly associated with patient's survival and could be used as an independent risk factor in prognostic prediction (Table 5). It is noteworthy that tumor cells could develop several strategies to escape NK-cell-mediated recognition in tumor microenvironment via immunoediting [18, 23]. It has been suggested that the interferon-γ-induced activation of JAK1 and JAK2 signaling pathway could suppress the tumor cell susceptibility to NK cells through up-regulation of PD-L1 expression [24]. In this context, the data from our and other groups have reported that the co-stimulatory molecule B7-H6 was over-expressed on cancer cells and binds to its receptor NKp30 on the NK cells, and thus leads to negative regulation of NK cells, which in-turn promotes cancer progression [25–29].

The macrophages, originating from bone marrow precursors as well as circulating and splenic monocytes, are also an important cohort of white blood cells, that have a phagocytic function and serve as the first-line of defense against pathogens, foreign substances and even cancer cells [30–32]. The macrophages infiltrating the tumor tissue are also known as tumor associated macrophages (TAMs), and have been suggested to play a critical role in the regulation of tumor microenvironment, and has physio-pathological affect on tumor initiation, intra-tumoral angiogenesis, immune suppression and tumor metastasis [30]. These TAMs usually polarize into two functional phenotypes, namely classically activated M1 and alternatively activated M2, in response to different microenvironment. The bulk of the literature suggested that M1-type TAMs have anti-tumor role, while M2-type TAMs promotes cancer progression, metastasis and intra-tumoral angiogenesis [18, 30, 33]. It has been demonstrated that in human lung cancer, the density of infiltrating macrophages was significantly associated with the density of micro-vessels and patients' prognosis [34]. In gastric cancer, the infiltration of polarized TAMs has been identified to be a novel independent prognostic factor and has been proposed to be combined with the TNM stage, to re-define the risk stratification system and to better stratify the patients' prognoses. The tipping of TAMs to an anti-tumoral phenotype has been speculated to be a promising therapeutic target for postoperative treatment [35]. Hu *et al*. [36] have reported that the M2-type TAMs in the tumor stroma conferred a poor prognosis in human pancreatic cancer. Another study by Atanasov *et al*. [37] demonstrated that the overall survival and recurrence free survival of the patients with hilar cholangiocarcinoma were significantly improved in patients with low infiltration of TAMs in tumor invasive front in contrast to those with a high infiltration.

Our current data showed that, in the patients with stage II+III esophageal cancer, the higher infiltration of TAMs in tumor nest was significantly associated with poorer survival, while the higher infiltration in tumor stroma was significantly associated with better survival. Consistent with above observation that the tumor microenvironment could polarize the macrophages, our data suggested that the macrophages infiltrating the tumor nest might be predominantly of M2 type, while the tumor stroma might be of M1 type. Moreover, based on the Cox model analysis, we found that the higher infiltrating density of CD68^+^ macrophages in tumor nest was associated with a poor survival, but in tumor stroma was significantly associated with better survival of stage II+III esophageal cancer patients. Further analysis of the combined prognostic value of the infiltrating density of CD68^+^ macrophages in tumor nest and stroma, showed varying degree of death risk ranging from high to medium to low. In addition, the analysis of CD57^+^ NK cells and CD68^+^ macrophages in combination also suggested the differential correlation with death risk.

In conclusion, our study showed that the densities of the infiltrating NK cells in tumor nest, and the infiltrating macrophages in both tumor nest and stroma, were significantly associated with patients' postoperative prognoses. The combination of infiltrating NK cells and macrophages both in tumor nest and stroma could also be used as important prognostic tools in predicting survival of the stage II+III esophageal cancer patients.

## MATERIALS AND METHODS

### Patient and tissues samples

Formalin-fixed, paraffin-embedded esophageal cancer tissue samples were collected from 138 patients, who underwent surgical resection, between November 2006 and March 2011 in our hospital. Among these patients, 102 were men, while 36 were women, and the median age at diagnosis was 59 years. No patients ever received pre-operative chemotherapy or radiotherapy and all tumor tissues were confirmed as the esophageal squamous cell carcinomas, using hematoxylin & eosin (H&E) staining after surgical resection. The tumor-node-metastasis (TNM) stages were assigned according to the American Joint Committee on cancer criteria [38]. The detailed clinical parameters of the patients are shown in Table 1. The protocols for the present study were approved by the ethics committee of the hospital.

### Immunohistochemistry

Formalin-fixed, paraffin-embedded tissues were cut into 3-mm-thick sections, and were then dewaxed in xylene, rehydrated and graded in ethanol solutions. Antigens were retrieved by heating the tissue sections at 100°C for 30 min in citrate (10 mmol/L, pH6.0) buffer. Next, the sections were immersed in a 0.3% hydrogen peroxide solution for 30 min to block endogenous peroxidase activity, and rinsed in phosphate buffered saline (PBS) for 5 min, blocked with 3% BSA at room temperature for 30 min, before incubating with anti-CD57 or anti-CD68 antibodies (Fuzhou Maxim Biotechnology, Fuzhou, China). Negative controls were processed by omitting the incubation with primary antibodies. The HRP-labeled goat anti mouse/rabbit secondary antibody (K500711, Dako, Glostrup, Denmark) was then used, according to the manufacturer's instruction. The diaminobenzene was used as a chromogen, and hematoxylin as the nuclear counterstain. The sections were then finally dehydrated, cleared and mounted.

### Evaluation of immunohistochemical staining

All slides were examined independently by two senior pathologists, who were not informed of patients' clinical parameters. The infiltrating immune cells in human esophageal cancer tissues were detected as described previously in our studies [8, 39, 40]. Briefly, the infiltrating CD57^+^ NK cells and CD68^+^ macrophages in the tumor nest were counted as follows: first, five areas with intense infiltration of either CD57^+^ NK cells or CD68^+^ macrophages, were selected at low magnification (×40). This was followed by counting and recording of these cells at high power field (HPF, ×200 magnification). Then, these infiltrating CD57^+^ NK cells and CD68^+^ macrophages in the tumor stroma were evaluated at low magnification (×40) and categorized based on the density as follows: Grade 0, scanty; Grade 1, moderate infiltration; Grade 2, abundant infiltration; Grade 3, massive infiltration. The group with Grade 0 and 1 constituted low infiltration group, while group with Grade 2 and 3 was defined as high infiltration group.

### Statistical analyses

All statistical analyses were performed by using SPSS 22.0 software (SPSS, Inc., Chicago, IL). Chi-square test was used to compare the disease-related factors in the patients with low and high infiltrating NK cells and macrophages in the tumor nest and stroma. Kaplan-Meier method and the log rank test were used for comparing survival curves. The Cox model was used to assess the hazard ratio (HR) with 95% CI (confidence interval), for association of the infiltrating densities of NK cells and macrophages with the patient death after adjusting for the potential confounders. The *P value* of less than 0.05 based on the two-sided test was considered to be statistically significant.
